# Safety and Outcomes of Thyroid Surgery: A High-Volume Center Case Series and the Role of Intraoperative Neuromonitoring in Preventing Complications

**DOI:** 10.3390/jcm14176077

**Published:** 2025-08-28

**Authors:** Mariarita Tarallo, Cecilia Carlino, Daniele Crocetti, Giuseppe Cavallaro, Andrea Polistena, Enrico Fiori, Paolo Sapienza, Marco Bononi

**Affiliations:** Department of Surgery, Policlinico “Umberto I”, Sapienza University of Rome, 00161 Rome, Italy; ceciliacarlinomd@gmail.com (C.C.); daniele.crocetti@uniroma1.it (D.C.); giuseppe.cavallaro@uniroma1.it (G.C.); andrea.polistena@uniroma1.it (A.P.); enrico.fiori@uniroma1.it (E.F.); paolo.sapienza@uniroma1.it (P.S.); marco.bononi@uniroma1.it (M.B.)

**Keywords:** thyroid surgery, intraoperative neuromonitoring (IONM), recurrent laryngeal nerve (RLN) palsy, hypoparathyroidism

## Abstract

**Background:** Thyroidectomy is one of the most performed endocrine operations worldwide; among the most significant and feared complications are hypoparathyroidism and recurrent laryngeal nerve (RLN) injury. The purpose of this study is to analyze clinical outcomes and complication rates in thyroid surgery performed at a single high-volume center, with a specific focus on the impact of intraoperative neuromonitoring (IONM) of the recurrent laryngeal nerve. **Methods:** A retrospective observational study was conducted on 1263 patients who underwent thyroid surgery between 2009 and 2024. Data on demographics, surgical procedures, and postoperative complications were collected. Outcomes were compared between the pre-IONM (2009–2017) and post-IONM (2018–2024) periods. Statistical analysis included descriptive measures, chi-square or Fisher’s exact tests, and Kaplan–Meier survival analysis with log-rank comparison. **Results:** Among the 1263 procedures, 76.7% were total thyroidectomies. The overall incidence of transient and permanent hypoparathyroidism was 2.37% and 0.79%, respectively. RLN injuries included 2 bilateral palsies (0.16%, pre-IONM only), 37 transient unilateral palsies (2.93%), and 10 permanent unilateral palsies (0.79%). After IONM introduction, the incidence of RLN injuries significantly decreased (*p* = 0.03), and no bilateral injuries were observed (*p* = 0.04). Kaplan–Meier analysis showed that 92% of transient RLN palsies resolved within 4 months, with significantly faster recovery in the post-IONM group (log-rank *p* = 0.02). **Conclusions:** Thyroid surgery in high-volume centers is associated with low complication rates. The implementation of IONM, particularly continuous monitoring, has significantly improved RLN preservation and enhanced recovery from transient injuries. These findings support the routine integration of IONM in thyroid surgery to maximize safety and functional outcomes.

## 1. Introduction

In the evolving landscape of surgical specialization, thyroid surgery has increasingly become the domain of dedicated endocrine surgery units. With an estimated 40,000 thyroid procedures performed annually in Italy and over 150,000 in the United States, thyroidectomy is one of the most commonly performed endocrine operations worldwide [1]. The indications span from benign nodular disease and Graves’ disease to thyroid malignancies [2], and while the procedure is generally safe in experienced hands, it is not without potentially serious complications.

Among the most significant and feared complications are hypoparathyroidism and recurrent laryngeal nerve (RLN) injury. A recent Italian multicenter study [3] established the incidence of hypoparathyroidism was 28.8%, including transient hypocalcemia (27.9%) and permanent hypocalcemia (0.9%), depending on surgical technique and institutional volume [4]. RLN injuries, which may result in dysphonia or even airway compromise in bilateral cases, have a reported incidence of 2–8% for temporary injury and 0.5–3% for permanent injury [5,6].

Centralization of thyroid surgery in high-volume centers has been shown to reduce the incidence of these complications. Studies suggest that high-volume surgeons (defined as performing > 50 thyroidectomies per year [2]) have significantly lower complication rates compared to low-volume surgeons [7]. Moreover, high-volume centers are more likely to have experienced multidisciplinary teams and advanced intraoperative technologies that contribute to safer outcomes [8].

One such advancement is intraoperative neuromonitoring (IONM), which has emerged as a useful adjunct to visual nerve identification during thyroidectomy [9]. IONM provides real-time functional feedback on RLN integrity, potentially allowing for early detection and prevention of nerve injury. There are two main forms of IONM: intermittent neuromonitoring, where the RLN is stimulated manually at defined steps of the operation, and continuous neuromonitoring, which delivers real-time, ongoing feedback throughout the procedure [10]. The use of the IONM has been widespread in Italy in recent years [11]. A growing body of literature supports the utility of IONM, particularly continuous monitoring, in reducing the incidence of nerve injuries and bilateral palsies, especially in high-risk or reoperative cases [5,12]. In addition to improving safety, IONM has educational value, enhancing the learning curve for less experienced surgeons. Recent meta-analyses and systematic reviews have further confirmed the role of IONM in reducing RLN injury rates [13,14,15].

The present study aims to evaluate the safety of thyroid surgery in a high-volume academic center over a 15-year period, with particular attention to the impact of IONM introduced in 2018 [16]. By analyzing complication rates and comparing pre- and post-IONM eras, we aim to provide data that further clarify the role of IONM in modern thyroid surgery and contribute to ongoing efforts to optimize patient outcomes.

## 2. Materials and Methods

A retrospective observational analysis was carried out on patients who underwent thyroid surgery at the Department of Surgery of the Policlinico Umberto I, Sapienza University of Rome, over the period between 2009 and 2024. The study population included all patients who underwent surgical treatment for either benign or malignant thyroid diseases. Patients were excluded from the analysis if their clinical records were incomplete or if adequate follow-up data were not available, ensuring the reliability of the collected outcomes.

Preoperative evaluation systematically involved thyroid function tests and high-resolution neck ultrasonography. When indicated, fine-needle aspiration cytology (FNAC) was performed to assess the nature of thyroid nodules. Furthermore, all patients underwent preoperative fibrolaryngoscopy to assess baseline vocal cord mobility and to document any pre-existing dysfunction.

Starting from 2018, intraoperative neuromonitoring (IONM) was routinely adopted in all thyroid surgeries performed at our institution [16]. A Nerve Integrity Monitor (NIM-Response) and electromyography (EMG) endotracheal tube (Medtronic, Jacksonville, FL, USA) were used for monitoring. This technological advancement aimed to enhance the identification and preservation of the recurrent laryngeal nerve, with the goal of reducing nerve-related complications. Procedures were performed using either intermittent or continuous IONM techniques. In all cases, intraoperative neuromonitoring was employed according to current international guidelines. Both intermittent IONM (I-IONM) and continuous IONM (C-IONM) were utilized depending on the specific case, device availability, and surgeon judgment. I-IONM was performed through repeated stimulations of the recurrent laryngeal nerve (RLN) using a handheld probe, with electromyographic (EMG) signals recorded via surface electrodes integrated into the endotracheal tube positioned near the vocal cords. This method supported RLN identification, especially in anatomically altered fields, and allowed for functional assessment before and after dissection. C-IONM, based on CMAP (compound muscle action potentials), involved the placement of an Automatic Periodic Stimulation (APS) electrode on the vagus nerve to allow continuous monitoring of RLN function in real time. This technique enabled the early detection of impending nerve injury and intraoperative assessment of functional recovery. Pre- and post-operative vocal cord mobility was assessed via laryngoscopy in all patients, and IONM steps followed the standard protocol proposed by the International Neural Monitoring Study Group [17]. Surgeries performed were total thyroidectomy, lobohistmectomy (elective or lobohisthmectomy performed in case of loss of signal from IONM during first lobectomy), and completion thyroidectomy (the surgical removal of the remnant thyroid tissue following procedures of less than total or near-TT).

Postoperative complications were categorized according to their duration. Complications that resolved within six months following surgery were classified as transient, whereas those persisting beyond six months were considered permanent. In the postoperative setting, all patients who exhibited clinical signs of vocal cord dysfunction underwent repeat fibrolaryngoscopic examination to objectively determine the presence or absence of vocal cord paresis or paralysis.

### Statistical Analysis

For the statistical analysis, descriptive statistics were used to summarize patient demographics, types of surgical procedures, and the incidence of postoperative complications. Continuous variables were presented as means with standard deviations, while categorical variables were expressed as counts and percentages. The chi-square test or Fisher’s exact test was employed to compare the incidence of complications between the pre-IONM and post-IONM periods. Kaplan–Meier survival analysis was applied to evaluate time-to-event outcomes, such as the resolution of transient complications over time. A log-rank test was used to compare survival curves. Statistical significance was set at a *p*-value of less than 0.05. All analyses were performed using statistical software such as SPSS (version 27.0) or equivalent tools.

## 3. Results

Between 2009 and 2024, a total of 1263 thyroid surgeries were performed at our institution. The majority of procedures were total thyroidectomies (969, 76.7%), followed by total thyroidectomy with lymphadenectomy (161, 12.7%), lobectomy with histhmectomy (86, 6.8%), and completion thyroidectomy (47, 3.7%) (Table 1).

Postoperative complications are summarized in Table 2.

Transient hypoparathyroidism occurred in 30 patients (2.37%), including 13 cases following total thyroidectomy, 13 after total thyroidectomy with lymphadenectomy, and 4 after completion thyroidectomy. Permanent hypoparathyroidism was recorded in 10 patients (0.79%): 3 following total thyroidectomy, 3 after total thyroidectomy with lymphadenectomy, and 4 after completion thyroidectomy.

Recurrent laryngeal nerve (RLN) injuries were documented in 49 cases overall. Bilateral RLN palsy occurred in two patients (0.16%), exclusively in the pre-IONM era. Transient unilateral RLN palsy was noted in 37 patients (2.93%), and permanent unilateral RLN palsy in 10 cases (0.79%).

**Figure 1:**
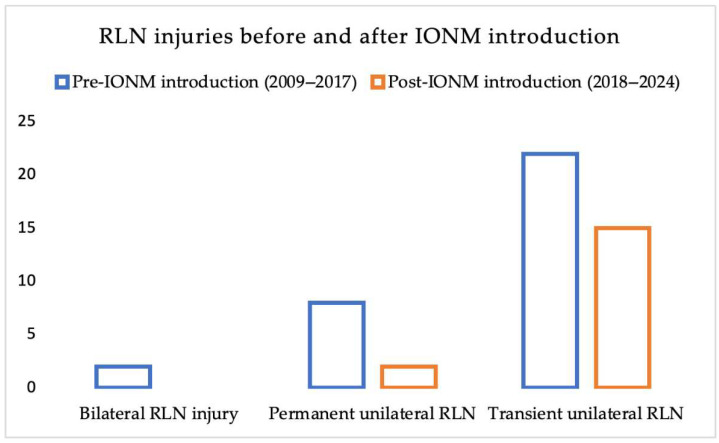
Incidence of recurrent laryngeal nerve (RLN) injuries before and after the introduction of intraoperative neuromonitoring (IONM). This figure illustrates the rates of RLN injuries in patients undergoing thyroid surgery between 2009 and 2024, divided into two periods: pre- and post-IONM (2018). A clear reduction is observed in both transient and permanent unilateral RLN palsies after IONM adoption. Notably, bilateral RLN injury was eliminated in the post-IONM era.

Since the introduction of intraoperative neuromonitoring (IONM) in 2018, 395 thyroid surgeries have been performed using this technique. Among these, 178 procedures (45%) employed intermittent IONM (I-IONM), while 217 (55%) used continuous IONM (C-IONM). In the I-IONM group, 4 cases of transient RLN palsy occurred, with no permanent injuries. In the C-IONM group, 2 cases of transient RLN palsy and 2 permanent injuries were observed. When comparing pre- and post-IONM periods (Table 3, Figure 1), we observed a reduction in transient RLN palsy from 22 to 15 cases and in permanent RLN palsy from 8 to 2 cases. Bilateral RLN injuries occurred only in the pre-IONM cohort. The reduction in overall RLN injuries was statistically significant (*p* = 0.03), and Fisher’s exact test confirmed a significant decrease in bilateral RLN injuries (*p* = 0.04), while the decrease in permanent unilateral palsies did not reach statistical significance (*p* = 0.11).

**Figure 2:**
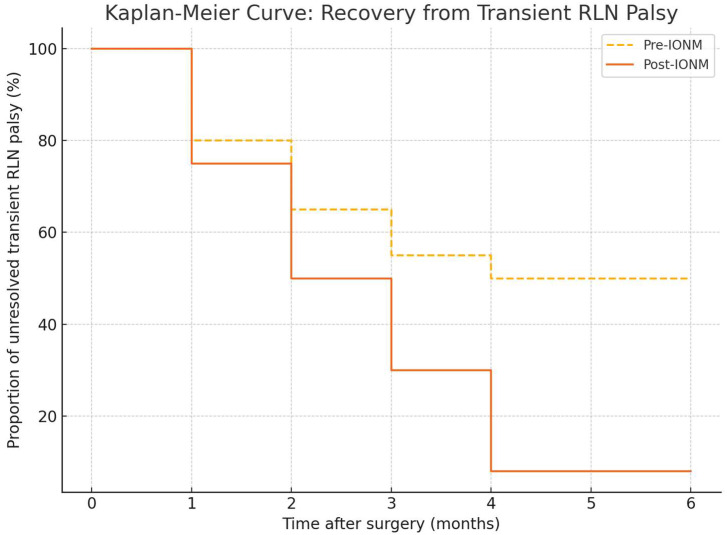
Kaplan–Meier analysis comparing recovery time from transient RLN palsy between pre-IONM and post-IONM cohorts. The post-IONM group showed significantly faster recovery (log-rank *p* = 0.02). The Kaplan–Meier curves presented clearly illustrate a more rapid decline in unresolved RLN palsy in the post-IONM group compared to the pre-IONM group. This visual finding is statistically supported by the log-rank test (*p* = 0.02), confirming a significantly faster recovery associated with IONM adoption.

Kaplan–Meier analysis of transient RLN palsy demonstrated that most cases resolved within four months postoperatively, with a 92% recovery rate. A significant difference in recovery time between pre- and post-IONM groups was observed (log-rank *p* = 0.02) (Figure 2). The median time to recovery was 3.2 months (IQR 2.5–4.0) in the pre-IONM group and 2.6 months (IQR 2.0–3.5) in the post-IONM group.

**Figure 3:**
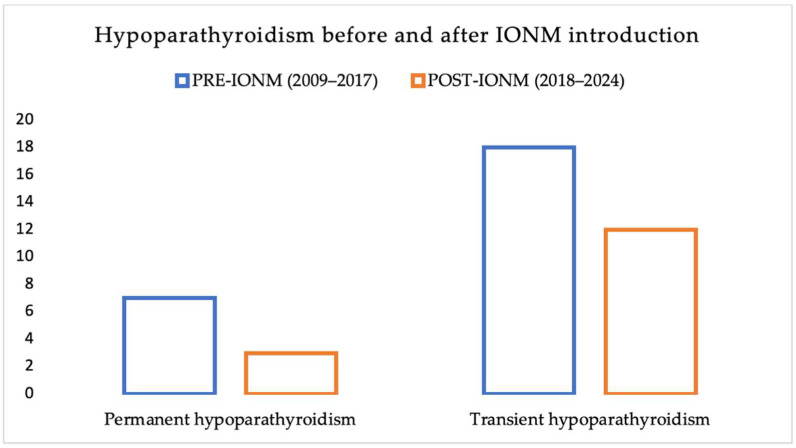
Incidence of Postoperative Hypoparathyroidism Before and After the Introduction of Intraoperative Neuromonitoring (IONM).

The incidence of hypoparathyroidism pre- and post-IONM introduction was assessed, although hypoparathyroidism is not a complication closely related to the management of RLN and the use of IONM (Table 4, Figure 3).

This figure presents the incidence of postoperative hypoparathyroidism (both transient and permanent) among patients undergoing thyroid surgery, stratified by the use of IONM. While IONM primarily targets nerve protection, the graph explores its potential impact on parathyroid preservation as an indirect effect of improved surgical precision.

### Sub-Analysis: Intermittent vs. Continuous IONM

A subgroup analysis was performed comparing patients undergoing intermittent intraoperative neuromonitoring (I-IONM, N = 178) and continuous intraoperative neuromonitoring (C-IONM, N = 217). Demographic, pathological, surgical, and outcome variables were compared between groups. The results are summarized in Table 5. Although some differences were noted in pathological distribution, no significant differences were observed in RLN palsy rates between the two subgroups. Given the limited sample size, these results should be considered exploratory.

## 4. Discussion

This study presents the outcomes of 1263 thyroid surgeries performed over a 15-year period in a high-volume endocrine surgery center, with a focus on the safety profile of the procedures and the impact of intraoperative neuromonitoring (IONM) on the incidence of major complications. The analysis confirms that thyroid surgery, when performed in specialized settings by experienced teams, is associated with low complication rates and excellent safety outcomes.

Hypoparathyroidism remains one of the most common postoperative complications following thyroidectomy. In our series, transient hypoparathyroidism occurred in 2.37% of patients and permanent hypoparathyroidism in 0.79%. These rates are significantly lower than those reported in the literature, particularly in Italian series, where transient hypoparathyroidism is described in 27.9% and permanent hypocalcemia [3]. Notably, most cases of hypoparathyroidism in our cohort occurred in patients undergoing total thyroidectomy, with or without lymphadenectomy, which is consistent with the known risk profile of more extensive procedures. These findings may reflect meticulous surgical dissection and systematic preservation of the parathyroid glands, alongside consistent perioperative management protocols. Although IONM is not directly designed to prevent hypocalcemia, recent studies suggest that improved precision during nerve identification may indirectly favor meticulous parathyroid preservation [15].

Recurrent laryngeal nerve (RLN) injury, both transient and permanent, is among the most feared complications in thyroid surgery due to its impact on phonation and, in bilateral cases, potential airway compromise. In our entire series, transient unilateral RLN palsy was observed in 2.93% of cases and permanent unilateral RLN palsy in 0.79%, aligning with published data reporting 2–8% for temporary injury and 0.5–3% for permanent injury [5,6]. Importantly, no cases of bilateral RLN injury occurred after the introduction of IONM in 2018. Before its implementation, two cases of bilateral RLN injury had been recorded (0.16%). The introduction of IONM, especially in a high-volume center, appears to be a pivotal factor in reducing RLN injury. Among 395 patients operated on after 2018 with IONM, we observed a lower incidence of both transient and permanent RLN palsy compared to the pre-IONM group. Specifically, permanent RLN palsy was recorded in 8 patients before 2018 and in only 2 patients after the adoption of IONM, with a statistically significant reduction. This effect was more pronounced in the group undergoing continuous IONM (C-IONM) (as we reported in our previous paper [16]), in which only 2 transient and 2 permanent RLN palsies were reported, compared to 4 transient cases in the intermittent IONM (I-IONM) subgroup [16]. Statistical analysis confirms these trends. The overall reduction in RLN injuries following IONM adoption reached statistical significance (*p* = 0.03), and bilateral injuries were completely eliminated post-IONM (*p* = 0.04). Time-to-event analysis with Kaplan–Meier curves showed that most transient RLN injuries resolved within four months, and patients in the post-IONM group recovered significantly faster (log-rank *p* = 0.02). The visual divergence between Kaplan–Meier curves (Figure 3) further supports this observation, with the log-rank test confirming that patients operated on in the post-IONM era had a significantly faster resolution of transient RLN palsy (*p* = 0.02). These findings support prior evidence from randomized trials and meta-analyses suggesting that IONM reduces the risk of nerve injury and aids in early intraoperative identification of nerve distress [18,19].

Our results are consistent with international guidelines, such as those from the International Neural Monitoring Study Group, which advocate for the use of IONM—particularly in high-risk or complex thyroid surgeries [20,21]. Furthermore, the structured implementation of pre- and postoperative laryngeal examination via fibrolaryngoscopy in all patients with suspected vocal cord dysfunction represents an additional strength of our perioperative strategy, ensuring accurate diagnosis and monitoring of RLN injuries. Beyond safety, IONM has been analyzed for cost-effectiveness. While some studies concluded it may not be cost-effective if only direct operative costs are considered [22], other analyses found it to be cost-effective in preventing bilateral RLN palsy during total thyroidectomy [23]. Moreover, recent data indicate that overall postoperative management costs related to RLN palsy are significantly reduced when IONM is used [24].

Our findings are consistent with recent systematic reviews and meta-analyses that further support the clinical value of IONM. Saxe et al. [13] reported a significant reduction in both transient and permanent RLN injuries with IONM, while Yang et al. [25] confirmed its protective role across a large cohort of patients.

The strengths of this study include the large sample size, the long observation period, and the consistency of surgical technique (surgeries were performed by the same surgeon with the same technique, thus eliminating a possible bias) and perioperative protocols within a single high-volume center. It should also be noted that the long observational period of 15 years may itself introduce potential confounders. Advances in surgical equipment, refinements in anesthetic management, and the natural progression of the surgical learning curve may have contributed to improved outcomes independently of IONM adoption. These factors should be acknowledged when interpreting the results. The detailed sub-analysis of IONM types (intermittent vs. continuous) also offers valuable insights into the relative effectiveness of each approach. However, the retrospective design introduces inherent limitations, including potential data incompleteness and selection bias. Furthermore, long-term functional outcomes and quality-of-life measures were not included in this analysis and represent an important area for future research.

In summary, this study reinforces the role of intraoperative neuromonitoring as an effective tool in improving the safety of thyroid surgery. In particular, continuous IONM may offer an additional layer of protection against RLN injuries and should be considered in high-risk cases or reoperations. As endocrine surgery continues to evolve, the integration of advanced monitoring technologies, standardized protocols, and multidisciplinary perioperative care will be crucial to further improving patient outcomes.

## 5. Conclusions

Thyroid surgery performed in a high-volume center using standardized techniques and intraoperative neuromonitoring is associated with low rates of major complications. The adoption of IONM, especially continuous monitoring, contributes significantly to the prevention of RLN injuries and enhances recovery from transient dysfunctions. These data support the integration of IONM into routine thyroid surgical practice, particularly in high-risk patients.

## Figures and Tables

**Table 1:** Thyroid surgeries.

Type of Procedure	Number (%)
Total thyroidectomy	969 (76.7%)
Total thyroidectomy + lymphadenectomy	161 (12.7%)
Lobectomy with histhmectomy	86 (6.8%)
Completion thyroidectomy	47 (3.7%)

Total thyroid surgeries performed between 2009 and 2024 (N = 1263). Type of procedure performed.

**Table 2:** Postoperative complications.

Complication	Total Cases (%)	*p*-Value *
Transient hypoparathyroidism	30 (2.37%)	–
Permanent hypoparathyroidism	10 (0.79%)	–
Bilateral RLN injury (pre-IONM only)	2 (0.16%)	0.04 (Fisher’s exact)
Transient unilateral RLN palsy	37 (2.93%)	0.32 (Chi-square)
Permanent unilateral RLN palsy	10 (0.79%)	0.27 (Fisher’s exact)

Postoperative complications following thyroid surgery. Study period: 2009–2024. IONM introduction: 2018. * Statistical comparisons refer to pre- vs. post-IONM period (see Table 3).

**Table 3:** RLN injuries before and after IONM introduction.

Complication	Pre-IONM (2009–2017)	Post-IONM (2018–2024)	*p*-Value *
Bilateral RLN injury	2	0	0.04 (Fisher’s exact)
Permanent unilateral RLN	8	2	0.11 (Fisher’s exact)
Transient unilateral RLN	22	15	0.32 (Chi-square test)
Overall RLN injuries			*p* = 0.03

Comparison of RLN injuries before and after IONM introduction. * Statistical significance set at *p* < 0.05.

**Table 4:** Hypoparathyroidism before and after IONM introduction.

Complication	Pre-IONM (2009–2017)	Post-IONM (2018–2024)	*p*-Value *
Permanent hypoparathyroidism	7	3	0.11 (Fisher’s exact)
Transient hypoparathyroidism	18	12	0.32 (Chi-square test)

Comparison of hypoparathyroidism before and after IONM introduction. * Statistical significance set at *p* < 0.05.

**Table 5:** Comparison between Intermittent (I-IONM) and Continuous (C-IONM) intraoperative neuromonitoring. Values are presented as counts (%) or mean ± SD. *p*-values refer to chi-square tests. (n.s.: not significant).

Variable	I-IONM (N = 178)	C-IONM (N = 217)	*p*-Value
Age (years)	57.7 ± 8	58.0 ± 8	n.s.
Sex (M:F)	45:133	70:147	0.1593
Pathology			
NMG	111 (62.36%)	107 (49.31%)	0.0127
TMG	26 (14.61%)	13 (5.99%)	0.0072
Thyroiditis	13 (7.30%)	21 (9.68%)	0.5113
Thyroid carcinoma	22 (12.36%)	55 (25.35%)	0.0018
Atypical adenoma	6 (3.37%)	21 (9.68%)	0.0231
Type of surgery			
Total thyroidectomy	122 (68.54%)	153 (70.51%)	0.7542
Lobectomy	56 (31.46%)	64 (29.49%)	0.7542
Operative time (min)	135.7 ± 25	146.3 ± 25	n.s.
Transient RLN palsy	9 (5.06%)	6 (2.76%)	0.3571
Permanent RLN palsy	0	2 (0.92%)	0.5675
Total RLN palsy	9 (5.06%)	8 (3.69%)	0.6758

## Data Availability

Data are available upon request with the permission of the last author.

## Abbreviations

The following abbreviations are used in this manuscript:

RLN	Recurrent laryngeal nerve
IONM	intraoperative neuromonitoring
I-IONM	intermittent intraoperative neuromonitoring
C-IONM	continuous intraoperative neuromonitoring

## References

[1] Patel K.N., Yip L., Lubitz C.C., Grubbs E.G., Miller B.S., Shen W., Angelos P., Chen H., Doherty G.M., Fahey T.J. (2020). The American Association of Endocrine Surgeons Guidelines for the Definitive Surgical Management of Thyroid Disease in Adults. Ann. Surg..

[2] Del Rio P., Polistena A., Chiofalo M.G., De Pasquale L., Dionigi G., Docimo G., Graceffa G., Iacobone M., Medas F., Pezzolla A. (2023). Management of surgical diseases of thyroid gland indications of the United Italian Society of Endocrine Surgery (SIUEC). Updates Surg..

[3] Puzziello A., Rosato L., Innaro N., Orlando G., Avenia N., Perigli G., Calò P.G., De Palma M. (2014). Hypocalcemia following thyroid surgery: Incidence and risk factors. A longitudinal multicenter study comprising 2631 patients. Endocrine.

[4] Edafe O., Antakia R., Laskar N., Uttley L., Balasubramanian S.P. (2014). Systematic review and meta-analysis of predictors of post-thyroidectomy hypocalcaemia. Br. J. Surg..

[5] Jonas J., Boskovic A. (2014). Intraoperative neuromonitoring (IONM) for recurrent laryngeal nerve protection: Comparison of intermittent and continuous nerve stimulation. Surg. Technol. Int..

[6] Malik R., Linos D. (2016). Intraoperative Neuromonitoring in Thyroid Surgery: A Systematic Review. World J. Surg..

[7] Ameri K., Kwon M., Watanabe A., Wiseman S.M. (2025). Thyroid cancer quality of care indicators: A scoping review. Am. J. Surg..

[8] Huston-Paterson H.H., Mao Y.V., Tseng C.H., Kim J., Chen D.W., Wu J.X., Yeh M.W. (2025). The Relationship Between Hospital Safety-Net Burden on Outcomes for High-Volume Thyroid Cancer Surgeons. Thyroid.

[9] Cirocchi R., Arezzo A., D’Andrea V., Abraha I., Popivanov G.I., Avenia N., Gerardi C., Henry B.M., Randolph J., Barczyñski M. (2019). Intraoperative neuromonitoring versus visual nerve identification for prevention of recurrent laryngeal nerve injury in adults undergoing thyroid surgery. Cochrane Database Syst. Rev..

[10] Anuwong A., Lavazza M., Kim H.Y., Wu C.W., Rausei S., Pappalardo V., Ferrari C.C., Inversini D., Leotta A., Biondi A. (2016). Recurrent laryngeal nerve management in thyroid surgery: Consequences of routine visualization, application of intermittent, standardized and continuous nerve monitoring. Updates Surg..

[11] Melcarne R., Docimo G., Aiello P.S.L., Andreani S., Avenia N., Basili G., Bellotti C., Bettini D., Biffoni M., Bononi M. (2025). Intraoperative nerve monitoring in thyroid and parathyroid surgery: A decade of Italian practice. Updates Surg..

[12] Schneider R., Randolph G.W., Sekulla C., Phelan E., Thanh P.N., Bucher M., Machens A., Dralle H., Lorenz K. (2013). Continuous intraoperative vagus nerve stimulation for identification of imminent recurrent laryngeal nerve injury. Head. Neck.

[13] Saxe A., Idris M., Gemechu J. (2024). Does the Use of Intraoperative Neuromonitoring during Thyroid and Parathyroid Surgery Reduce the Incidence of Recurrent Laryngeal Nerve Injuries? A Systematic Review and Meta-Analysis. Diagnostics.

[14] Yao L., Li J., Li M., Lin C., Hui X., Tamilselvan D., Kandi M., Sreekanta A., Makhdami N., Ali D.S. (2022). Parathyroid Hormone Therapy for Managing Chronic Hypoparathyroidism: A Systematic Review and Meta-Analysis. J. Bone Miner. Res..

[15] Wojtczak B., Sutkowska-Stępień K., Głód M., Kaliszewski K., Sutkowski K., Barczyński M. (2024). Current Knowledge on the Use of Neuromonitoring in Thyroid Surgery. Biomedicines.

[16] Cirillo B., Brachini G., Cavallaro G., Tarallo M., Carlino C., Duranti G., Zambon M., Mingoli A., Simonelli L., Bononi M. (2025). The Application of Intermittent Intraoperative Neuromonitoring (I-IONM) and Continuous Intraoperative Neuromonitoring (C-IONM) During Thyroid Surgery: A Single-Center Study. J. Clin. Med..

[17] Randolph G.W., Dralle H., Abdullah H., Barczynski M., Bellantone R., Brauckhoff M., Carnaille B., Cherenko S., Chiang F.Y., Dionigi G. (2011). Electrophysiologic recurrent laryngeal nerve monitoring during thyroid and parathyroid surgery: International standards guideline statement. Laryngoscope.

[18] Pisanu A., Porceddu G., Podda M., Cois A., Uccheddu A. (2014). Systematic review with meta-analysis of studies comparing intraoperative neuromonitoring of recurrent laryngeal nerves versus visualization alone during thyroidectomy. J. Surg. Res..

[19] Wong K.P., Au K.P., Lam S., Lang B.H. (2017). Lessons Learned After 1000 Cases of Transcutaneous Laryngeal Ultrasound (TLUSG) with Laryngoscopic Validation: Is There a Role of TLUSG in Patients Indicated for Laryngoscopic Examination Before Thyroidectomy?. Thyroid.

[20] Schneider R., Randolph G.W., Dionigi G., Wu C.W., Barczynski M., Chiang F.Y., Al-Quaryshi Z., Angelos P., Brauckhoff K., Cernea C.R. (2018). International neural monitoring study group guideline 2018 part I: Staging bilateral thyroid surgery with monitoring loss of signal. Laryngoscope.

[21] Wu C.W., Dionigi G., Barczynski M., Chiang F.Y., Dralle H., Schneider R., Al-Quaryshi Z., Angelos P., Brauckhoff K., Brooks J.A. (2018). International neuromonitoring study group guidelines 2018: Part II: Optimal recurrent laryngeal nerve management for invasive thyroid cancer-incorporation of surgical, laryngeal, and neural electrophysiologic data. Laryngoscope.

[22] Tae K. (2019). Cost-effectiveness of intraoperative neural monitoring in thyroid surgery: Comment on “Analyzing cost-effectiveness of neural-monitoring in recurrent laryngeal nerve recovery course in thyroid surgery”. Gland. Surg..

[23] Al-Qurayshi Z., Kandil E., Randolph G.W. (2017). Cost-effectiveness of intraoperative nerve monitoring in avoidance of bilateral recurrent laryngeal nerve injury in patients undergoing total thyroidectomy. Br. J. Surg..

[24] Kang I.K., Bae J.S., Kim J.S., Kim K. (2024). Cost-effectiveness of intraoperative neural monitoring of recurrent laryngeal nerves in thyroid lobectomy for papillary thyroid carcinoma. Ann. Surg. Treat. Res..

[25] Yang S., Zhou L., Lu Z., Ma B., Ji Q., Wang Y. (2017). Systematic review with meta-analysis of intraoperative neuromonitoring during thyroidectomy. Int. J. Surg..

